# Gestational Diabetes Mellitus and Polycystic Ovary Syndrome: A Proposed Intergenerational Metabolic Continuum Within the DOHaD Framework

**DOI:** 10.3390/healthcare14142204

**Published:** 2026-07-21

**Authors:** Miroslava Gojnić, Stefan Dugalić, Katarina Ivanović, Miloš Milinčić, Maja Macura

**Affiliations:** 1 Clinic for Gynecology and Obstetrics, University Clinical Centre of Serbia, 11000 Belgrade, Serbia; miroslavagojnicdugalic@yahoo.com (M.G.); ikatarina.1996@gmail.com (K.I.); milosmilincic@gmail.com (M.M.); maja_macura@live.com (M.M.); 2 Faculty of Medicine, University of Belgrade, 11000 Belgrade, Serbia

**Keywords:** gestational diabetes mellitus, polycystic ovary syndrome, insulin resistance, fetal programming, Developmental Origins of Health and Disease, intergenerational metabolic risk, metabolic programming, women’s health

## Abstract

**Background**: Gestational diabetes mellitus (GDM) and polycystic ovary syndrome (PCOS) are clinically distinct disorders that share important metabolic features. This Perspective proposes a non-causal intergenerational framework linking maternal PCOS-related metabolic vulnerability, susceptibility to GDM, intrauterine metabolic exposure, and later reproductive-metabolic risk in female offspring. **Methods**: A narrative synthesis of evidence from reproductive endocrinology, obstetrics, diabetology, developmental biology, and public health was undertaken. The strength of evidence supporting individual components of the proposed continuum was qualitatively appraised. Separately, aggregated surveillance data from Belgrade for 2015–2024 were used as a registry-based illustration of ICD-10 O24.4-coded GDM diagnoses. No formal time-series analysis, causal modeling, or forecasting was performed. **Results**: Evidence was strongest for the increased risk of GDM among women with PCOS and for the association between intrauterine exposure to maternal diabetes and later offspring metabolic susceptibility. Evidence linking maternal PCOS with offspring metabolic vulnerability was less definitive, while direct evidence that GDM exposure leads to clinically diagnosed PCOS in female offspring remained weak and indirect. Registry-recorded GDM rates varied across years, but this variability may reflect differences in screening, diagnostic criteria, coding, reporting completeness, and healthcare access rather than true changes in prevalence. **Conclusions**: The proposed GDM–PCOS continuum is a hypothesis-generating and probabilistic framework, not an established causal pathway. Its clinical value lies in connecting prevention opportunities before conception, during pregnancy, after delivery, and across the life course. Longitudinal maternal–offspring studies are required to test the proposed relationships.

## 1. Introduction

Gestational diabetes mellitus (GDM) is a common metabolic complication of pregnancy and an important marker of future maternal and offspring cardiometabolic risk [1,2,3,4]. Pregnancy may function as a physiological “stress test” that reveals pre-existing insulin resistance, limited pancreatic β-cell reserve, and susceptibility to later type 2 diabetes mellitus [1,3,4]. However, the recorded burden of GDM is influenced not only by underlying metabolic risk but also by screening strategies, diagnostic criteria, healthcare access, coding practices, and reporting completeness [1,2,3].

Polycystic ovary syndrome (PCOS) is a heterogeneous endocrine disorder characterized by ovulatory dysfunction, hyperandrogenism, and polycystic ovarian morphology [5,6]. Beyond its reproductive manifestations, PCOS is associated with insulin resistance, compensatory hyperinsulinemia, dyslipidemia, adipose tissue dysfunction, and an increased lifetime risk of impaired glucose tolerance and type 2 diabetes [5,6,7]. The severity of these metabolic abnormalities varies according to PCOS phenotype, body mass index, age, ethnicity, lifestyle, and genetic susceptibility.

Women with PCOS have an increased risk of GDM. A systematic review and meta-analysis of 104 studies and 106,690 pregnancies reported higher odds of GDM and other adverse pregnancy outcomes among women with PCOS, including in analyses matched for maternal age and body mass index [8]. A large population-based study similarly found an approximately twofold higher adjusted risk of GDM after accounting for obesity, age, race, socioeconomic characteristics, assisted reproductive technology, and pre-existing metabolic disease [9]. Nevertheless, the magnitude of this association remains heterogeneous and may be modified by PCOS phenotype, adiposity, maternal age, ethnicity, fertility treatment, socioeconomic conditions, and antenatal screening practices.

GDM and PCOS are clinically distinct conditions, but they may represent different manifestations of overlapping reproductive and metabolic vulnerability across the female life course. Women with PCOS may enter pregnancy with insulin resistance, hyperinsulinemia, excess adiposity, or limited β-cell reserve, increasing their susceptibility to GDM when physiological pregnancy-related insulin resistance develops [5,6,7,8,9]. Conversely, GDM may reveal a previously unrecognized metabolic phenotype. This overlap provides a rationale for considering the two conditions within a broader life-course framework without implying that they are equivalent or causally interchangeable.

The Developmental Origins of Health and Disease (DOHaD) framework provides a basis for examining how maternal metabolic exposures during pregnancy may influence long-term health in offspring [10,11,12]. Maternal hyperglycemia, obesity, insulin resistance, inflammation, and altered placental signaling may affect fetal growth, endocrine regulation, energy metabolism, and developmental programming [10,11,12,13,14]. Intrauterine exposure to maternal diabetes has been associated with later obesity, impaired glucose regulation, type 2 diabetes, and other cardiometabolic outcomes in offspring [13,14,15]. These associations may reflect both intrauterine effects and shared genetic, familial, socioeconomic, and postnatal determinants.

Developmental mechanisms have also been proposed in PCOS. Experimental studies suggest that prenatal androgenic and metabolic disturbances may influence ovarian development, hypothalamic–pituitary–ovarian regulation, insulin sensitivity, and adiposity in offspring [16,17]. Human population-based evidence indicates that maternal PCOS or anovulatory infertility is associated with increased obesity in offspring and possibly diabetes in female offspring during later adolescence or early adulthood [18]. Epigenetic regulation and germline transmission have also been proposed as potential contributors to the familial clustering of PCOS-related traits [19].

The strength of evidence differs across the proposed pathway. The association between PCOS and subsequent GDM is comparatively well supported [8,9], as is the relationship between intrauterine exposure to maternal diabetes and later offspring metabolic vulnerability [13,14,15]. Evidence linking maternal PCOS with offspring metabolic and reproductive outcomes is less definitive and remains predominantly observational [16,17,18,19]. Direct evidence that maternal GDM independently leads to clinically diagnosed PCOS in female offspring is currently weak and indirect.

The proposed intergenerational continuum should therefore be interpreted as a non-deterministic and hypothesis-generating framework. Maternal body mass index, age, PCOS phenotype, ethnicity, socioeconomic conditions, fertility treatment, healthcare access, paternal characteristics, and the postnatal environment may act as confounders, mediators, or effect modifiers. Only a proportion of affected women and offspring would be expected to follow the complete sequence.

Aggregated registry data can illustrate the recorded burden of GDM and identify weaknesses in metabolic surveillance during pregnancy. They cannot, however, directly test the proposed GDM–PCOS continuum without individual-level information on maternal PCOS phenotype, metabolic status, treatment, offspring exposure, and long-term reproductive outcomes. The registry component of this Perspective is therefore interpreted as a contextual epidemiological illustration rather than as empirical validation of the proposed framework.

### Novel Contributions of This Perspective

Existing DOHaD models have described associations between maternal metabolic dysfunction and long-term offspring health, while previous research has separately examined the increased risk of GDM in women with PCOS, the developmental origins of PCOS, and the cardiometabolic consequences of intrauterine exposure to maternal diabetes [8,9,13,14,15,16,17,18,19]. The novelty of the present Perspective therefore does not lie in presenting any single biological mechanism or association as previously unrecognized. Instead, its contribution is the integration of these previously fragmented lines of evidence into a unified, clinically oriented, and testable intergenerational sequence.

First, the proposed framework explicitly connects preconception PCOS and metabolic vulnerability with susceptibility to GDM; GDM with an altered intrauterine endocrine-metabolic environment; and fetal metabolic programming with later susceptibility to obesity, insulin resistance, and PCOS-related reproductive-metabolic phenotypes in female offspring. It further proposes that this vulnerability may increase GDM risk when affected offspring subsequently enter pregnancy. This complete PCOS → GDM → intrauterine exposure → offspring reproductive-metabolic vulnerability → subsequent GDM sequence has not generally been articulated as a single life-course model.

Second, this Perspective distinguishes established associations from more speculative components of the model. The PCOS–GDM association and the relationship between GDM exposure and later offspring metabolic risk are supported by comparatively stronger evidence [8,9,13,14,15]. By contrast, the pathway from maternal GDM exposure to the later development of clinically defined PCOS in female offspring remains insufficiently demonstrated and represents a specific research gap rather than a confirmed causal relationship. This distinction prevents biological plausibility from being interpreted as proof of causality.

Third, the proposed framework incorporates maternal body mass index, age, ethnicity, socioeconomic status, PCOS phenotype, lifestyle, fertility treatment, healthcare access, diagnostic practices, paternal characteristics, and the postnatal environment as potential confounders, mediators, or effect modifiers rather than treating them only as limitations. Their position within the framework emphasizes that intergenerational transmission is shaped by the interaction of biological susceptibility with social, environmental, familial, and healthcare-system determinants.

Finally, the framework translates the proposed continuum into coordinated opportunities for prevention before conception, during pregnancy, after delivery, and during the long-term follow-up of mothers and offspring. It also defines a testable research agenda requiring longitudinal studies that integrate maternal PCOS phenotype and metabolic status, GDM screening and treatment, placental and epigenetic biomarkers, paternal and postnatal characteristics, and reproductive and metabolic outcomes in female offspring.

The present Perspective therefore has two deliberately separated objectives. The first is to formulate a clinically oriented and testable conceptual framework linking maternal PCOS, susceptibility to GDM, intrauterine metabolic exposure, and later reproductive-metabolic vulnerability in female offspring. The second is to present aggregated registry-recorded GDM diagnoses as a contextual epidemiological illustration of the recorded burden of GDM and the challenges of metabolic surveillance during pregnancy. The registry-based component is not intended to validate the proposed intergenerational pathway. By clearly separating these two components and distinguishing established evidence from hypothetical links, this Perspective aims to identify clinically relevant prevention opportunities and specific priorities for future research.

## 2. Methods

### 2.1. Design and Scope of the Perspective

This article was designed as a narrative, hypothesis-generating Perspective examining the potential relationship between gestational diabetes mellitus (GDM), polycystic ovary syndrome (PCOS), and developmental programming within the Developmental Origins of Health and Disease (DOHaD) framework. The primary objective was not to establish a direct causal association between GDM and PCOS, but to formulate a biologically and clinically plausible model linking maternal metabolic vulnerability, pregnancy-related metabolic exposure, and long-term reproductive and metabolic susceptibility in female offspring.

The manuscript comprises two analytically distinct components. The first is a conceptual component that integrates evidence concerning shared metabolic and endocrine mechanisms, including insulin resistance, compensatory hyperinsulinemia, androgen excess, adipose tissue dysfunction, chronic low-grade inflammation, placental metabolic signaling, and fetal endocrine-metabolic programming. This component provides the basis for the proposed intergenerational GDM–PCOS continuum.

The second component is a registry-based epidemiological illustration using aggregated public health surveillance data on diabetes in pregnancy coded according to the International Classification of Diseases, 10th Revision (ICD-10) [20]. Its purpose is to demonstrate variability in registry-recorded GDM diagnoses and to contextualize the public health and surveillance environment in which pregnancy-related metabolic disorders are identified. It was not designed to test, confirm, or quantify the proposed intergenerational GDM–PCOS pathway.

The two components are therefore complementary at the level of clinical and public health interpretation but are not analytically linked. The conceptual synthesis addresses biological plausibility and identifies testable intergenerational hypotheses, whereas the registry-based component addresses diagnostic recording, surveillance variability, and healthcare-system context. Because the surveillance dataset contained no individual-level information on PCOS status, maternal phenotype, offspring outcomes, or relevant confounding variables, no direct association between the empirical data and the proposed mechanistic pathway could be evaluated.

Given the aggregated nature of the available surveillance data, no causal inference, patient-level risk modeling, time-series modeling, or adjustment for confounding was performed. The population-level findings were interpreted as descriptive indicators of registry-recorded diagnostic variability rather than as estimates of true disease prevalence or evidence supporting a causal continuum.

### 2.2. Literature Basis and Conceptual Synthesis

The conceptual component of this Perspective was based on a targeted narrative synthesis rather than a systematic review or meta-analysis. Relevant literature was identified through iterative searches of PubMed/MEDLINE, supplemented by manual screening of the reference lists of key international guidelines, systematic reviews, meta-analyses, and landmark articles. The literature search was conducted during manuscript preparation and was updated during the revision process.

Search terms were used individually and in different combinations and included “gestational diabetes mellitus”, “GDM”, “polycystic ovary syndrome”, “PCOS”, “Developmental Origins of Health and Disease”, “DOHaD”, “fetal programming”, “developmental programming”, “intergenerational”, “offspring”, “insulin resistance”, “hyperinsulinemia”, “androgen excess”, “adipose tissue dysfunction”, “inflammation”, “placenta”, “epigenetic programming”, and “pregnancy outcomes”.

Titles and abstracts were screened for direct relevance to one or more components of the proposed framework. Full-text articles were reviewed when their relevance could not be determined from the title and abstract alone. Priority was given to international clinical guidelines, recent systematic reviews and meta-analyses, large population-based or longitudinal cohort studies, and mechanistic or experimental studies that directly informed specific components of the proposed continuum. Older landmark studies were retained when they provided foundational biological or clinical concepts that were not adequately represented by more recent publications.

Studies were considered relevant when they addressed at least one of the following domains:

1. insulin resistance and compensatory hyperinsulinemia as shared features of GDM and PCOS;
2. androgen excess and altered ovarian endocrine regulation;
3. obesity, adipose tissue dysfunction, and chronic low-grade inflammation;
4. placental metabolic signaling, nutrient transfer, and fetal exposure;
5. developmental or epigenetic programming of metabolic and reproductive outcomes;
6. pregnancy outcomes in women with PCOS;
7. long-term metabolic or reproductive outcomes in offspring exposed to maternal GDM or PCOS.

Articles were not retained when they did not directly contribute to the biological plausibility, clinical interpretation, critical appraisal, or testable components of the proposed framework. No restrictions were imposed according to study design when mechanistic evidence was required; however, evidence from experimental models was interpreted separately from evidence derived from human observational studies.

Study selection was not performed in duplicate, and no prospectively registered protocol, predefined eligibility framework, exhaustive database search, formal study-level risk-of-bias assessment, or quantitative evidence synthesis was undertaken. The qualitative classifications of evidence strength presented in Section Critical Appraisal of the Supporting Evidence and Table 1 therefore reflect the authors’ appraisal of the consistency, directness, methodological robustness, and clinical relevance of the available literature rather than a formal GRADE assessment.

The purpose of the literature synthesis was to integrate evidence from reproductive endocrinology, obstetrics, diabetology, developmental biology, and public health into a coherent hypothesis-generating framework. Accordingly, the proposed GDM–PCOS continuum should be interpreted as a conceptual model requiring validation in prospective longitudinal studies with individual-level maternal and offspring data.

### 2.3. Registry-Based Epidemiological Illustration

To provide a public health context for pregnancy-related metabolic surveillance, aggregated data were obtained from the Belgrade Institute of Public Health for the period from 1 January 2015 to 31 December 2024. These data were collected through routine health reporting and comprised annual counts of pregnancy-related diagnostic categories coded according to ICD-10 [20].

The available dataset included pre-existing insulin-dependent diabetes mellitus complicating pregnancy (O24.0), pre-existing non-insulin-dependent diabetes mellitus complicating pregnancy (O24.1), diabetes mellitus related to malnutrition complicating pregnancy (O24.2), other specified pre-existing diabetes mellitus complicating pregnancy (O24.3), gestational diabetes mellitus (O24.4), unspecified diabetes mellitus in pregnancy (O24.9), excessive weight gain during pregnancy (O26.0), and obesity complicating pregnancy, childbirth, and the puerperium (O99.2). GDM was operationally identified through ICD-10 code O24.4.

Annual numbers of deliveries were obtained from the Health Statistical Yearbooks of the Republic of Serbia published by the Institute of Public Health of Serbia “Dr Milan Jovanović Batut” and from the corresponding official annual reports for 2023 and 2024 [21,22,23,24,25,26,27,28,29,30]. Registry-recorded GDM diagnosis rates were calculated as the annual number of recorded O24.4 diagnoses per 1000 deliveries. The aggregated dataset used for these descriptive calculations is provided as Supplementary File S1.

The resulting measures represent registry-recorded diagnostic rates rather than epidemiological prevalence or incidence estimates. The analysis was descriptive and did not include formal time-series analysis, tests for temporal trend, change-point analysis, or adjustment for changes in population structure, screening coverage, diagnostic criteria, healthcare access, or reporting practices.

The aggregate dataset did not permit assessment of record-level completeness or missingness. No individual patient records were available, and independent validation of ICD-10 coding against laboratory findings, oral glucose tolerance test results, medical records, or treatment data could not be performed. Therefore, the extent of under-recording, duplicate recording, diagnostic misclassification, or variation in reporting completeness could not be quantified.

The dataset contained no individual-level information on maternal age, body mass index, parity, ethnicity, socioeconomic status, PCOS diagnosis or phenotype, fertility treatment, gestational weight gain, glucose-testing strategy, treatment exposure, glycemic control, pregnancy outcome, or offspring health. Consequently, associations between GDM, PCOS, maternal metabolic characteristics, and intergenerational outcomes could not be examined.

The registry-based component was therefore included exclusively as an epidemiological illustration of how frequently GDM was recorded within the available surveillance system and how the recorded diagnostic burden varied across calendar years. It should not be interpreted as empirical validation of the conceptual GDM–PCOS continuum.

No projections of future GDM diagnosis rates were performed. The available dataset consisted of a limited number of annual aggregated observations and did not contain the individual-level demographic, metabolic, clinical, or healthcare-system variables required for a validated forecasting model. In addition, marked interannual variability and the possibility of changes in screening, coding, and reporting practices precluded a defensible assumption that the observed pattern would continue into future years. Accordingly, the registry component was restricted to a descriptive epidemiological illustration of the 2015–2024 observation period.

### 2.4. Ethical Considerations

The study used aggregated, de-identified public health surveillance data and did not involve individual patient-level information. The protocol was reviewed and approved by the Ethics Committee of the University Clinical Centre of Serbia, Faculty of Medicine, University of Belgrade, approval number 1880/6. Individual informed consent was not required because the analysis was based exclusively on aggregated surveillance data in accordance with institutional and national regulations.

## 3. Shared Metabolic and Endocrine Pathways Linking GDM and PCOS

### 3.1. Insulin Resistance, Hyperinsulinemia, and Androgen Excess

Insulin resistance represents the principal metabolic overlap between gestational diabetes mellitus (GDM) and polycystic ovary syndrome (PCOS). In PCOS, impaired insulin action can involve multiple tissues and is observed across both higher-weight and lean phenotypes, although its severity varies according to body mass index, PCOS phenotype, genetic susceptibility, and lifestyle factors [31,32,33]. During normal pregnancy, insulin sensitivity progressively decreases, whereas pancreatic β-cell secretion increases to maintain maternal glucose homeostasis. GDM develops when this compensatory response is insufficient to overcome pregnancy-related insulin resistance [34,35].

Hyperinsulinemia provides an important mechanistic connection between metabolic and reproductive dysfunction. In women with PCOS, insulin acts synergistically with luteinizing hormone to stimulate androgen production in ovarian theca cells [36,37]. Insulin also reduces hepatic synthesis of sex hormone-binding globulin, thereby increasing circulating free androgen concentrations and potentially aggravating anovulation and other reproductive manifestations of PCOS [37,38].

Insulin resistance is not uniformly present across all PCOS phenotypes, and its contribution to GDM risk cannot be separated completely from obesity, maternal age, ethnicity, family history, and other metabolic characteristics [7,31,32,33,39]. Women with PCOS may nevertheless enter pregnancy with reduced metabolic reserve and a greater likelihood of failing to compensate for physiological pregnancy-related insulin resistance. Conversely, GDM may reveal an underlying metabolic vulnerability that was clinically less apparent before pregnancy.

Recent systematic reviews, meta-analyses, and large population-based studies consistently demonstrate an increased occurrence of GDM among women with PCOS, while also showing substantial heterogeneity according to diagnostic criteria, age, body mass index, study setting, and other clinical factors [8,9,40,41]. This overlap supports a life-course interpretation of GDM and PCOS without implying that the disorders are equivalent or that PCOS necessarily causes GDM.

### 3.2. Adipose Tissue Dysfunction, Inflammation, and Phenotypic Heterogeneity

Adipose tissue dysfunction may amplify the metabolic abnormalities shared by GDM and PCOS. Visceral adiposity, adipocyte hypertrophy, altered adipokine secretion, and chronic low-grade inflammation are associated with impaired insulin signaling and greater reproductive and metabolic risk [39,42,43,44,45]. Reduced adiponectin concentrations and increased inflammatory mediators, including tumor necrosis factor-alpha and interleukin-6, may contribute to systemic insulin resistance, although these abnormalities are neither specific to GDM nor PCOS [42,43,44,45].

Obesity is an important risk amplifier but does not account for all metabolic abnormalities observed in PCOS. Insulin resistance may also occur in women with lean PCOS phenotypes, whereas not all women with obesity develop PCOS or GDM. Accordingly, maternal body mass index should be interpreted as one component of a broader metabolic phenotype that also includes fat distribution, androgen status, glucose regulation, family history, ethnicity, lifestyle, and socioeconomic conditions.

Longitudinal evidence further indicates that body mass index trajectories and PCOS may jointly influence the subsequent risk of GDM [46]. However, the contribution of adiposity is complex: it may precede PCOS, intensify insulin resistance and hyperandrogenism, mediate part of the association between PCOS and GDM, or modify the severity of both conditions.

This heterogeneity has important implications for the proposed continuum. The risk of GDM is unlikely to be uniform across all women with PCOS, and developmental consequences are similarly likely to differ according to the severity of maternal metabolic dysfunction, glycemic control, treatment exposure, gestational weight gain, and the postnatal environment. These characteristics may function as confounders, mediators, or effect modifiers rather than as background variables.

### 3.3. Placental Signaling and the Intrauterine Metabolic Environment

The placenta represents the principal interface through which maternal metabolic disturbances may influence fetal development. It regulates nutrient transfer, endocrine signaling, inflammatory responses, and fetal growth. Maternal hyperglycemia, obesity, insulin resistance, and inflammation may alter placental structure and function, including nutrient transport, oxidative stress, endocrine signaling, and inflammatory pathways [47,48,49].

Maternal glucose crosses the placenta, whereas maternal insulin does not cross in clinically relevant amounts. Increased fetal glucose exposure stimulates fetal insulin secretion, which may promote fetal growth, adiposity, and metabolic adaptation [34,47,50]. These effects depend on the timing and severity of maternal hyperglycemia, placental function, gestational weight gain, treatment, and glycemic control.

Placental alterations may influence fetal development independently of maternal glucose concentrations alone. However, placental findings vary across studies, and it remains difficult to distinguish causal mediators from adaptive responses or markers of maternal metabolic dysfunction. Placental function should therefore be considered a potential intermediary rather than a proven causal link between maternal GDM and later reproductive-endocrine outcomes.

Developmental and epigenetic mechanisms, including altered DNA methylation, histone regulation, and non-coding RNA expression, have been proposed as pathways through which intrauterine metabolic exposures may exert persistent effects after birth [51,52,53]. At present, these mechanisms provide biological plausibility but should not be interpreted as established mediators of clinically defined PCOS in female offspring. It often remains uncertain whether observed epigenetic alterations represent causes, mediators, consequences, or biomarkers of the underlying metabolic state.

Table 1 provides a critical synthesis of the principal components of the proposed continuum, including the qualitative strength of supporting evidence, clinical relevance, and major remaining uncertainties.

**Table 1:** Critical synthesis of evidence supporting the proposed intergenerational GDM–PCOS continuum.

Component of the Proposed Continuum	Principal Supporting Evidence	Qualitative Strength of Evidence	Clinical Relevance	Main Uncertainties and Limitations
PCOS → increased susceptibility to GDM	Meta-analyses and large population-based studies consistently demonstrate a higher occurrence of GDM among women with PCOS. The association remains evident in several analyses adjusted or matched for maternal age and body mass index [8,9,40,41].	Relatively strong	PCOS may identify women who could benefit from preconception metabolic assessment, early glucose evaluation, and individualized antenatal surveillance.	Evidence is predominantly observational. Risk estimates vary according to PCOS phenotype, body mass index, maternal age, ethnicity, diagnostic criteria, fertility treatment, screening strategy, and study quality. PCOS-associated metabolic traits may explain part of the observed risk.
Shared insulin resistance and compensatory hyperinsulinemia	Insulin resistance is common in PCOS and contributes to GDM when pregnancy-related insulin resistance exceeds pancreatic β-cell compensatory capacity. Hyperinsulinemia may also stimulate ovarian androgen production and reduce hepatic sex hormone-binding globulin synthesis [31,32,33,34,35,36,37,38].	Relatively strong for shared metabolic biology; moderate as a specific intergenerational mediator	Supports metabolic assessment before conception, weight management, glucose surveillance, and prevention of type 2 diabetes.	Insulin resistance is heterogeneous and is not equally expressed across all PCOS phenotypes. Its contribution is difficult to separate from obesity, genetic susceptibility, age, and lifestyle.
GDM → altered intrauterine metabolic environment	Maternal hyperglycemia increases transplacental glucose exposure and fetal insulin secretion. GDM is also associated with altered placental nutrient transport, inflammatory signaling, oxidative stress, and fetal growth regulation [34,47,48,49,50].	Relatively strong for maternal–fetal metabolic exposure	Timely diagnosis and treatment of GDM can reduce short-term pregnancy complications and may limit fetal exposure to an adverse metabolic environment.	Exposure varies according to the timing and severity of hyperglycemia, maternal obesity, gestational weight gain, placental function, treatment, and glycemic control. These factors are not consistently measured across studies.
GDM exposure → offspring obesity and metabolic susceptibility	Epidemiological and experimental studies associate intrauterine exposure to maternal diabetes with later adiposity, impaired glucose metabolism, diabetes, and broader cardiometabolic risk in offspring [13,14,15].	Moderate to relatively strong	Supports long-term preventive strategies for mothers and offspring and reinforces pregnancy as an opportunity for intergenerational prevention.	Shared genetics, maternal obesity, paternal metabolic characteristics, socioeconomic conditions, diet, and the postnatal environment may contribute to the observed associations. The independent effect of intrauterine hyperglycemia remains difficult to quantify.
Maternal PCOS → offspring metabolic and reproductive vulnerability	Population-based studies suggest increased obesity and metabolic abnormalities in offspring of women with PCOS. Experimental models demonstrate that prenatal androgenic and metabolic disturbances can produce reproductive and metabolic PCOS-like traits [16,17,18,19].	Moderate for general metabolic susceptibility; limited for clinically defined PCOS in human offspring	May justify prospective research and surveillance of offspring metabolic health, particularly when maternal PCOS coexists with obesity or diabetes.	Human evidence is observational and may be affected by exposure misclassification, maternal obesity, GDM, genetic inheritance, fertility treatment, and shared family environment. Experimental exposures do not fully reproduce heterogeneous human PCOS.
GDM exposure → PCOS in female offspring	Hyperglycemia, fetal hyperinsulinemia, inflammation, altered placental signaling, and developmental programming provide biological plausibility for later reproductive-endocrine vulnerability. Direct longitudinal evidence of clinically diagnosed PCOS in adult daughters remains scarce [12,13,14,15,16,17,18,19].	Weak and indirect	Identifies a specific research priority rather than supporting an immediate change in clinical practice.	Most studies assess offspring obesity, glucose intolerance, or experimental PCOS-like traits rather than standardized PCOS diagnosis after reproductive maturation. Causality has not been established.
Epigenetic and developmental programming mechanisms	Altered DNA methylation, histone regulation, and non-coding RNA expression have been reported in PCOS- and GDM-related maternal, placental, and offspring tissues. Experimental models support developmental and potential transgenerational effects [14,19,51,52,53].	Moderate for biological plausibility; limited for proven mediation in humans	May eventually support biomarkers and individualized prevention but currently remains primarily research-oriented.	Findings vary by tissue, timing, population, and analytical platform. It is often unclear whether epigenetic alterations are causes, mediators, consequences, or biomarkers of metabolic dysfunction.
BMI, age, ethnicity, socioeconomic conditions, PCOS phenotype, lifestyle, and healthcare access	These factors influence PCOS expression, GDM risk, diagnostic ascertainment, treatment, and offspring outcomes and may modify several stages of the proposed continuum [6,7,8,9,39,40,41,46].	Strong as determinants of risk and heterogeneity; uncertain at each individual pathway step	Risk assessment should be individualized rather than based only on the presence of PCOS or GDM. Prevention should include metabolic, behavioral, social, and healthcare-system factors.	Their roles may differ as confounders, mediators, or effect modifiers. They are incompletely measured and inconsistently adjusted for in many studies.

**Note:** The strength-of-evidence categories are qualitative and reflect the consistency, directness, and methodological robustness of the available literature. They do not represent a formal GRADE assessment because this Perspective was based on a narrative rather than systematic review methodology. The proposed continuum is non-deterministic, probabilistic, and hypothesis-generating. **Abbreviations:** BMI, body mass index; GDM, gestational diabetes mellitus; PCOS, polycystic ovary syndrome.

## 4. DOHaD and Intergenerational Metabolic Programming

The Developmental Origins of Health and Disease (DOHaD) framework proposes that environmental, endocrine, and metabolic exposures during critical periods of development may influence long-term disease susceptibility. Within this framework, pregnancy represents a biologically sensitive period during which maternal metabolic status may affect fetal growth, organ development, endocrine regulation, and later metabolic health [10,11,12].

For pregnancies complicated by gestational diabetes mellitus (GDM), the strongest evidence concerns the association between intrauterine exposure to maternal diabetes and later offspring metabolic vulnerability. Maternal hyperglycemia increases fetal glucose exposure and fetal insulin secretion, while maternal obesity, inflammation, placental function, gestational weight gain, and treatment may further modify the intrauterine environment. Offspring exposed to GDM have an increased risk of adiposity, impaired glucose regulation, type 2 diabetes, and other cardiometabolic outcomes [13,14,15]. However, these associations cannot be attributed exclusively to intrauterine hyperglycemia because shared genetics, family lifestyle, socioeconomic conditions, paternal metabolic characteristics, and the postnatal environment may also contribute.

Developmental programming has also been proposed as one component of PCOS pathogenesis. Experimental models demonstrate that prenatal androgenic and metabolic disturbances can influence ovarian function, hypothalamic–pituitary–ovarian regulation, insulin sensitivity, and adiposity in offspring [16,17,19]. Human observational studies suggest that offspring of women with PCOS may have increased metabolic vulnerability, but direct evidence linking maternal metabolic exposure with a standardized diagnosis of PCOS in adult daughters remains limited [18,19]. Experimental PCOS-like phenotypes should therefore not be considered equivalent to clinically diagnosed human PCOS.

The proposed intergenerational GDM–PCOS continuum integrates these findings into a probabilistic sequence. Women with PCOS may enter pregnancy with insulin resistance or other metabolic characteristics that increase susceptibility to GDM. GDM may subsequently expose the fetus to an altered endocrine-metabolic environment associated with later metabolic vulnerability. In genetically or environmentally susceptible female offspring, this vulnerability could contribute to PCOS-related reproductive and metabolic phenotypes and potentially increase GDM susceptibility during a later pregnancy. The complete sequence remains a testable hypothesis rather than an established causal pathway.

Several factors may modify each stage of the proposed continuum, including maternal body mass index, age, PCOS phenotype, ethnicity, genetic background, fertility treatment, severity and timing of hyperglycemia, glycemic control, socioeconomic conditions, healthcare access, paternal metabolic characteristics, and postnatal lifestyle. These factors may function as confounders, mediators, or effect modifiers and may explain why only a proportion of women and offspring would be expected to follow the proposed sequence.

The clinical value of the framework lies in identifying connected opportunities for prevention across the female life course rather than in predicting an inevitable intergenerational outcome. Preconception metabolic assessment in women with PCOS, appropriate GDM screening and treatment, postpartum glucose surveillance, and long-term health promotion for mothers and offspring may reduce cumulative metabolic risk. Longitudinal studies combining maternal phenotype, metabolic exposure, treatment, placental and epigenetic markers, paternal and postnatal characteristics, and offspring reproductive outcomes are required to determine which components of the proposed continuum are causal and clinically actionable.

**Figure 1:**
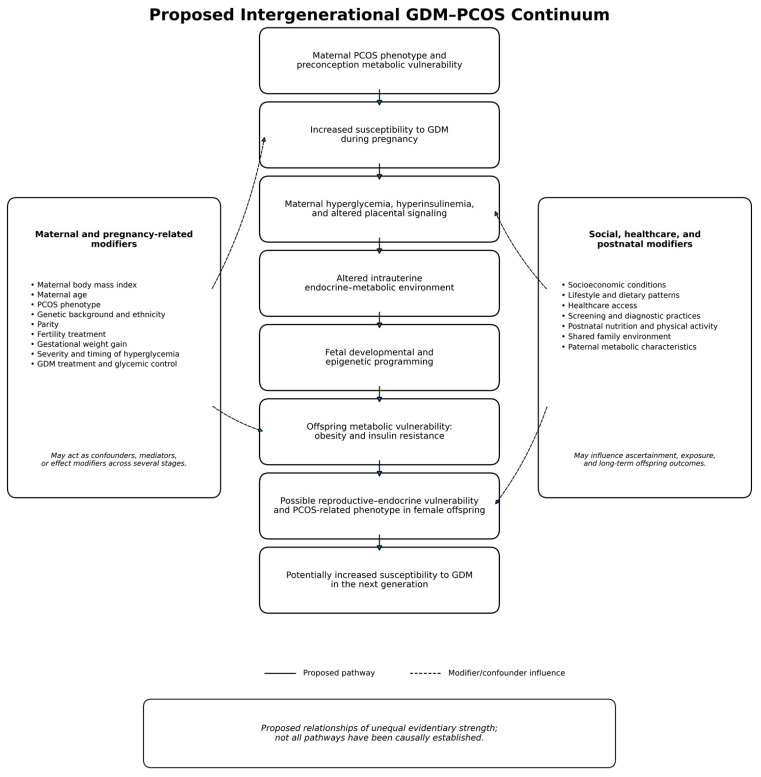
Non-causal conceptual model of the proposed intergenerational GDM–PCOS continuum. Maternal PCOS phenotype and preconception metabolic vulnerability may increase susceptibility to gestational diabetes mellitus (GDM). Pregnancy-related hyperglycemia, hyperinsulinemia, and altered placental signaling may contribute to an adverse intrauterine endocrine-metabolic environment and developmental programming. These exposures may be associated with later metabolic vulnerability and, potentially, PCOS-related reproductive-endocrine phenotypes in female offspring. Maternal body mass index, age, PCOS phenotype, genetic background, ethnicity, fertility treatment, socioeconomic conditions, healthcare access, treatment exposure, paternal metabolic characteristics, and the postnatal environment may act as confounders, mediators, or effect modifiers at different stages. The model is probabilistic and hypothesis-generating. Arrows indicate proposed relationships and do not imply that each step has been causally established.

The proposed non-causal and probabilistic intergenerational GDM–PCOS framework, together with its principal biological, clinical, social, and healthcare-related modifiers, is illustrated in Figure 1.

### Critical Appraisal of the Supporting Evidence

The evidence supporting the proposed intergenerational GDM–PCOS continuum is heterogeneous and differs substantially across its individual components. Accordingly, the continuum should not be interpreted as a single pathway supported by a uniform level of evidence. Rather, it integrates associations ranging from comparatively well-established clinical relationships to mechanistically plausible but insufficiently demonstrated intergenerational links. Because the present article is a narrative Perspective rather than a systematic review, the following classifications represent a qualitative appraisal of the consistency, directness, and methodological strength of the available evidence rather than a formal certainty-of-evidence assessment.

The association between maternal PCOS and subsequent GDM represents the most strongly supported component of the proposed continuum. Large population-based studies and recent systematic reviews and meta-analyses consistently indicate that women with PCOS have an increased risk of GDM compared with women without PCOS [8,9,40,41]. Importantly, the association has remained evident in analyses matched or adjusted for maternal age and body mass index, suggesting that PCOS-related risk cannot be attributed exclusively to obesity or older maternal age [8,9]. On this basis, the evidence supporting the PCOS → GDM component can be considered relatively strong, although it remains predominantly observational and therefore does not establish that PCOS itself directly causes GDM.

Substantial heterogeneity remains within this evidence base. Reported GDM occurrence among women with PCOS varies widely across studies, and maternal age, geographic region, study quality, sample size, body mass index, ethnicity, assisted reproductive technology use, screening strategy, and diagnostic criteria contribute to differences in risk estimates [40,41]. PCOS itself comprises several reproductive and metabolic phenotypes, and women with hyperandrogenic, anovulatory, obese, or insulin-resistant phenotypes may not share the same pregnancy-related metabolic risk. Furthermore, differences between the Rotterdam, National Institutes of Health, and other diagnostic definitions influence the characteristics of populations classified as having PCOS. Therefore, the PCOS → GDM association is consistent at the population level but should not be interpreted as uniform across all PCOS phenotypes or clinical settings.

The relationship between intrauterine exposure to GDM and later metabolic vulnerability in offspring is supported by a substantial body of epidemiological and experimental evidence [13,14,15]. Offspring exposed to maternal diabetes have an increased likelihood of childhood adiposity, impaired glucose metabolism, type 2 diabetes, and other cardiometabolic outcomes. Experimental and molecular studies also provide plausible mechanisms involving fetal hyperinsulinemia, altered placental nutrient signaling, adipose tissue programming, and epigenetic regulation. The evidence supporting the GDM → offspring metabolic susceptibility component can therefore be considered moderate to relatively strong.

Nevertheless, the direct intrauterine contribution of GDM is difficult to separate from shared familial and environmental determinants. Maternal obesity, genetic susceptibility, dietary patterns, socioeconomic conditions, ethnicity, paternal metabolic characteristics, and the postnatal environment may contribute both to maternal GDM and to later metabolic outcomes in offspring. Differences in the severity and timing of maternal hyperglycemia, treatment exposure, gestational weight gain, and postnatal follow-up further complicate comparisons between studies. Thus, the association with offspring metabolic risk is well supported, but the proportion attributable specifically to intrauterine hyperglycemia remains uncertain.

Evidence linking maternal PCOS with metabolic and reproductive vulnerability in offspring is emerging but less definitive. Population-based data indicate associations between maternal PCOS or anovulatory infertility and obesity in offspring, with some evidence of increased diabetes risk in female offspring during later adolescence and early adulthood [18]. Experimental models additionally demonstrate that prenatal androgen and metabolic exposure can produce reproductive and metabolic PCOS-like traits in subsequent generations [16,17,19]. These findings support developmental plausibility but do not establish an equivalent causal pathway in humans.

The interpretation of maternal PCOS–offspring associations is limited by exposure misclassification, incomplete characterization of PCOS phenotypes, residual confounding by maternal obesity and diabetes, and the inability of registry-based studies to distinguish genetic inheritance from intrauterine programming and shared postnatal environment. Experimental models permit more direct evaluation of biological mechanisms but frequently use androgen or anti-Müllerian hormone exposures that may not fully reproduce the heterogeneous endocrine-metabolic environment of human PCOS. Evidence for the maternal PCOS → offspring metabolic or reproductive vulnerability component should therefore be considered moderate for general metabolic susceptibility but limited for the later development of a clinically defined PCOS phenotype.

The least established component of the proposed continuum is the direct pathway from maternal GDM exposure to PCOS in female offspring. Current evidence supports the possibility that maternal hyperglycemia, fetal hyperinsulinemia, inflammation, placental dysfunction, and epigenetic changes may influence later reproductive and metabolic regulation. However, direct prospective human studies linking well-characterized maternal GDM exposure with a subsequent standardized diagnosis of PCOS in adult female offspring remain limited. Most available evidence concerns general offspring obesity, glucose intolerance, cardiometabolic risk, or experimental PCOS-like traits rather than clinically diagnosed PCOS. Consequently, the GDM → offspring PCOS component should be classified as biologically plausible but currently supported by weak and indirect evidence.

The interaction between concurrent PCOS and GDM also requires cautious interpretation. Although both conditions are independently associated with adverse pregnancy outcomes, recent register-based evidence did not demonstrate that the coexistence of PCOS and GDM consistently produces additional risk beyond that associated with GDM alone for a range of maternal and neonatal outcomes [54]. This finding argues against assuming a simple additive or synergistic relationship and further supports a model in which phenotype, obesity, treatment, and other clinical modifiers determine individual risk.

Epigenetic evidence represents another area of biological plausibility accompanied by substantial uncertainty. Altered DNA methylation, histone regulation, and non-coding RNA expression have been reported in maternal, placental, and offspring tissues in relation to GDM and PCOS [14,19,51,52,53]. However, findings differ according to tissue, timing of sampling, study population, analytical platform, and adjustment for obesity and other metabolic factors. In many studies, it remains uncertain whether the reported epigenetic alterations represent causes, mediators, consequences, or biomarkers of the underlying metabolic state. Epigenetic mechanisms should therefore be presented as candidate pathways rather than established mediators of intergenerational transmission.

Overall, the proposed continuum is supported most strongly at its PCOS → GDM and GDM → offspring metabolic susceptibility components. Evidence for maternal PCOS → offspring metabolic vulnerability is emerging, whereas evidence for GDM → clinically defined PCOS in female offspring remains insufficient. The conceptual value of the model lies not in implying that all links have been proven, but in identifying the specific transition between offspring metabolic programming and later reproductive PCOS phenotype as a priority for longitudinal investigation. The qualitative strength, clinical relevance, and principal uncertainties associated with each component are summarized in Table 1.

## 5. Registry-Based Epidemiological Illustration of GDM Recording

To provide a population-level context for metabolic surveillance during pregnancy, aggregated public health data from the Belgrade Institute of Public Health were examined for the period 2015–2024. The dataset included diagnoses related to gestational diabetes mellitus, pre-existing diabetes complicating pregnancy, unspecified diabetes in pregnancy, excessive gestational weight gain, and maternal obesity, coded according to the International Classification of Diseases, 10th Revision [20].

**Figure 2:**
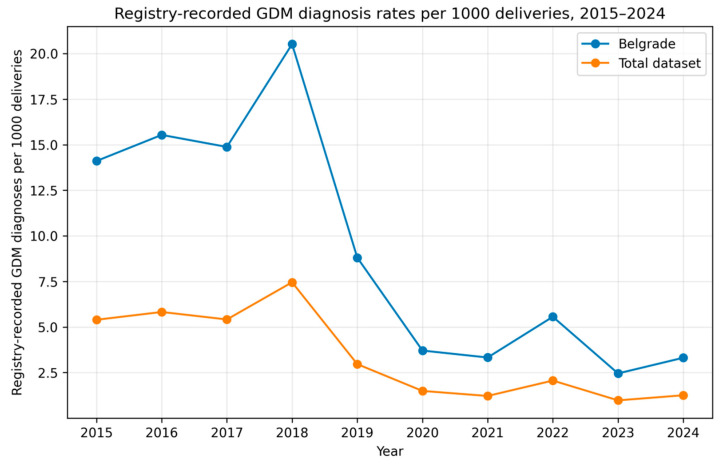
Registry-recorded gestational diabetes mellitus diagnosis rates per 1000 deliveries, 2015–2024. Annual rates were calculated using the number of ICD–10 O24.4 diagnoses and annual delivery counts as approximate denominators [20,21,22,23,24,25,26,27,28,29,30]. The figure demonstrates interannual variability in registry-recorded diagnoses, including the highest recorded value in 2018 and lower recorded values after 2019. The figure is descriptive and non-causal; it does not represent a formal epidemiological trend analysis or provide evidence of changes in true GDM prevalence, population metabolic risk, or the proposed intergenerational GDM–PCOS pathway.

Gestational diabetes mellitus, recorded under ICD-10 code O24.4, was the most frequently documented diabetes-specific category during the observation period. Annual numbers of recorded diagnoses, delivery denominators, and calculated registry-recorded diagnosis rates are presented in Table 2 and Figure 2. Delivery denominators were obtained from official national health statistical publications and annual reports [21,22,23,24,25,26,27,28,29,30].

The registry-recorded GDM diagnosis rate in Belgrade was 14.11 per 1000 deliveries in 2015 and reached its highest recorded value of 20.52 per 1000 deliveries in 2018. Lower recorded values were observed after 2019. A comparable pattern of interannual variability was present in the total dataset, although the absolute recorded rates were lower.

These observations should be described as registry-recorded diagnostic variability rather than as evidence of an epidemiological increase followed by a true decline in GDM occurrence. The available data do not permit determination of whether differences between calendar years resulted from changes in underlying metabolic risk, screening coverage, diagnostic thresholds, referral pathways, coding procedures, data submission, or healthcare utilization.

In particular, the higher recorded values during 2015–2018 and the lower values after 2019 should not be interpreted as evidence that population metabolic risk initially increased and subsequently decreased. Such an interpretation would be unsupported because no individual-level metabolic, demographic, clinical, or screening data were available.

Several healthcare-system and policy-related mechanisms may have influenced the recorded counts. These include changes in the proportion of pregnant women screened for GDM, use of universal versus risk-based screening, availability and completion of oral glucose tolerance testing, referral between primary, secondary, and tertiary care, laboratory capacity, diagnostic coding conventions, electronic reporting procedures, and institutional data-submission practices. Differences in any of these processes could alter the number of recorded O24.4 diagnoses independently of true changes in GDM occurrence.

An additional source of uncertainty is the potential evolution or non-uniform application of GDM screening and diagnostic criteria during the 2015–2024 period. Although the WHO/IADPSG 75-g oral glucose tolerance test thresholds were available internationally during this period [2], the aggregated surveillance dataset and the accompanying annual reports did not document which screening strategy or diagnostic criteria were applied in Serbia, in Belgrade, or at individual reporting institutions in each calendar year. We were therefore unable to determine whether the WHO/IADPSG approach, alternative one- or two-step procedures, risk-based screening, or institution-specific protocols were used consistently over time. Any change in screening coverage, test selection, glucose thresholds, or implementation across levels of care could alter the number of O24.4 diagnoses independently of a biological change in GDM occurrence. This uncertainty is considered a potential source of ascertainment and classification bias and limits direct comparison of annual registry-recorded rates.

Additional caution is required when interpreting observations from the COVID-19 period. Disruptions in antenatal attendance, laboratory services, oral glucose tolerance testing, referral pathways, and routine reporting may have affected diagnostic ascertainment. International evidence indicates that GDM screening procedures were modified during the pandemic, including greater reliance on fasting plasma glucose, random plasma glucose, or glycated hemoglobin in place of standard oral glucose tolerance testing [55]. Because the present dataset contained no information on the tests used during individual calendar years, the magnitude and direction of this potential bias could not be quantified.

Accordingly, the observed variation is most appropriately interpreted as a combined product of disease recording, diagnostic ascertainment, and healthcare-system performance. The available data cannot distinguish between a change in the number of women with GDM and a change in the probability that GDM was screened for, diagnosed, coded, and reported.

The observed registry-recorded rates were not extrapolated beyond 2024. Future GDM burden will depend on changes in maternal age, body mass index, population composition, screening coverage, diagnostic criteria, healthcare access, coding procedures, and data-reporting completeness. Because these determinants were unavailable in the aggregated dataset, projecting the observed values into future years would require unsupported assumptions and could create a misleading impression of forecasting precision.

Future projections should therefore be based on validated population-level models incorporating demographic and metabolic predictors, explicitly defined assumptions, alternative scenarios, and uncertainty intervals. The present Perspective does not estimate future GDM prevalence or future registry-recorded diagnosis rates.

Although these data do not permit evaluation of individual-level relationships between GDM, PCOS, maternal metabolic characteristics, and offspring outcomes, they provide a contextual illustration of the continuing public health importance of metabolic disorders during pregnancy. More importantly, they demonstrate the limitations of surveillance systems that report diagnostic counts without simultaneously documenting screening practices, diagnostic tests, relevant maternal characteristics, treatment, and pregnancy outcomes.

### Relationship of the Registry-Based Illustration to the Conceptual Framework

The registry-based findings do not provide a direct analytical test of the proposed intergenerational GDM–PCOS continuum. The available data contain no information on PCOS prevalence, maternal PCOS phenotype, preconception insulin resistance, pregnancy treatment, offspring exposure, or later reproductive and metabolic outcomes. Therefore, neither the PCOS → GDM pathway nor the GDM → offspring PCOS susceptibility pathway can be evaluated using these data.

The registry component contributes to the conceptual discussion in a more limited and indirect manner. First, it illustrates that GDM represents a routinely recorded pregnancy-related metabolic diagnosis with potential relevance for preventive healthcare. Second, the marked variability in recorded rates demonstrates the difficulty of monitoring metabolic disease burden when screening, diagnostic testing, coding, and reporting practices are not captured together with diagnostic counts.

From a policy perspective, the findings support the need for standardized GDM screening and coding procedures, consistent reporting denominators, documentation of the diagnostic test used, and improved linkage between obstetric, endocrine, primary care, and public health datasets. Future surveillance systems should, where legally and ethically permissible, include maternal age, body mass index, PCOS status and phenotype, parity, ethnicity or migration background, socioeconomic indicators, fertility treatment, gestational weight gain, glycemic treatment, and pregnancy outcomes.

Longitudinal linkage with offspring health and reproductive data would be required before the proposed intergenerational relationships could be examined empirically. Without such linkage, annual GDM diagnostic counts cannot demonstrate fetal programming, later PCOS susceptibility, or transmission of metabolic vulnerability across generations.

The registry-based component should therefore be viewed as a contextual epidemiological illustration that identifies surveillance limitations and healthcare-system priorities. It does not demonstrate temporal changes in biological susceptibility and does not provide evidence of causality between GDM, PCOS, and offspring outcomes.

The available observations also do not support quantitative forecasting of future GDM rates. Although increasing maternal age, obesity, insulin resistance, and the metabolic burden associated with PCOS could plausibly contribute to a greater future need for GDM prevention and surveillance, the direction and magnitude of registry-recorded rates will also depend on screening policies, diagnostic thresholds, healthcare utilization, coding practices, and reporting completeness. Instead of forecasting future burden, the registry component identifies the variables and surveillance infrastructure that future population-level models would need to incorporate.

## 6. Clinical, Public Health, and Research Implications

The proposed intergenerational GDM–PCOS continuum should not be interpreted as a basis for a new diagnostic category or as evidence that all women with PCOS, GDM, or intrauterine GDM exposure will follow the proposed sequence. Its principal clinical value lies in organizing existing prevention opportunities across the female life course and distinguishing components that are already relevant to clinical care from those that remain research hypotheses.

### 6.1. Preconception and Antenatal Risk Identification

PCOS may serve as an early marker of reproductive and metabolic vulnerability before pregnancy. Women with PCOS have an increased risk of GDM and other adverse pregnancy outcomes, although the magnitude of this risk varies according to body mass index, maternal age, PCOS phenotype, ethnicity, fertility treatment, family history, and other metabolic characteristics [6,8,9,40,41]. Risk assessment should therefore be individualized rather than based on PCOS status alone.

Preconception care should include assessment of body weight and weight trajectory, blood pressure, glucose regulation, lifestyle factors, family history of diabetes, and other relevant cardiometabolic risks [6]. Where clinically indicated, optimization of weight, nutrition, physical activity, and pre-existing metabolic disease should be addressed before conception.

During pregnancy, PCOS status should be documented and considered together with established GDM risk factors when planning metabolic surveillance [6,8]. However, the proposed framework does not support unvalidated PCOS-specific screening or treatment protocols. Rather, it reinforces the implementation of existing evidence-based recommendations and the need for coordinated obstetric, endocrine, and primary care.

The coexistence of PCOS and GDM should also not automatically be interpreted as indicating a uniformly more severe pregnancy phenotype. Available evidence suggests that individual risk depends on maternal body mass index, glycemic severity, treatment, PCOS phenotype, and other clinical modifiers, and that concurrent PCOS and GDM do not necessarily produce additional risk beyond that associated with GDM alone for every maternal or neonatal outcome [54].

### 6.2. Postpartum and Life-Course Follow-Up

GDM should be regarded as a sentinel event indicating increased long-term maternal metabolic risk rather than as a condition that necessarily resolves without further consequences after delivery. Women with a history of GDM remain at substantially increased risk of type 2 diabetes and other cardiometabolic disorders [3,4].

Postpartum glucose assessment and continued periodic screening for dysglycemia are therefore important components of preventive care [3]. Lifestyle support, weight management, nutritional counseling, promotion of physical activity, and appropriate referral should be integrated into longitudinal follow-up pathways rather than limited to an isolated short-term postpartum visit.

A life-course approach requires continuity between gynecological, obstetric, endocrine, and primary care services. Women with PCOS may enter pregnancy with pre-existing metabolic vulnerability, whereas women with prior GDM may later present with impaired glucose regulation, type 2 diabetes, or other cardiometabolic abnormalities. Linking these stages of care may improve early identification of risk and reduce missed preventive opportunities.

The proposed continuum does not imply that prevention at one stage will necessarily interrupt all later components of the model. Nevertheless, optimization of maternal metabolic health before conception, appropriate recognition and treatment of GDM, and structured postpartum follow-up represent established and clinically actionable opportunities, independent of whether the complete intergenerational pathway is ultimately confirmed.

### 6.3. Offspring Health, Surveillance, and Research Priorities

Evidence is stronger for an association between intrauterine exposure to GDM and later general metabolic susceptibility, including obesity, impaired glucose regulation, and diabetes, than for the subsequent development of clinically defined PCOS [13,14,15]. Maternal PCOS has also been associated with metabolic vulnerability in offspring, although these observations remain susceptible to confounding by maternal obesity, diabetes, genetics, and the shared family environment [18].

The available evidence does not justify screening female offspring for PCOS solely because they were exposed to maternal GDM. Preventive recommendations for offspring should therefore focus on healthy growth, balanced nutrition, physical activity, and avoidance of excess adiposity rather than disease-specific surveillance that is not yet supported by direct evidence.

Testing the proposed continuum will require prospective longitudinal studies linking:

1. maternal PCOS diagnosis and phenotype;
2. preconception body mass index and metabolic status;
3. GDM screening method, diagnostic criteria, timing, and severity;
4. gestational weight gain and glycemic treatment;
5. placental, inflammatory, and epigenetic markers;
6. paternal metabolic characteristics and the shared family environment;
7. postnatal nutrition, growth, physical activity, and socioeconomic exposures;
8. standardized reproductive and metabolic outcomes in female offspring after reproductive maturation.

Future studies should distinguish clinically diagnosed PCOS from experimental PCOS-like traits and should include adequate adjustment for maternal age, body mass index, ethnicity, socioeconomic conditions, fertility treatment, paternal characteristics, and postnatal lifestyle. Whenever possible, sibling-comparison designs, negative-control analyses, genetic methods, and longitudinal measurement of potential mediators may help distinguish intrauterine effects from shared familial susceptibility.

From a public health perspective, surveillance systems should move beyond annual diagnostic counts toward standardized and linkable maternal–offspring data. Documentation of the diagnostic test used, screening coverage, maternal PCOS status, treatment exposure, pregnancy outcomes, and relevant demographic and metabolic characteristics would permit more reliable evaluation of disease burden and, eventually, intergenerational hypotheses.

Until such data are available, the registry component of this Perspective should be interpreted as an illustration of surveillance needs and healthcare-system variability rather than as evidence validating the proposed biological pathway.

## 7. Limitations

### 7.1. Limitations of the Narrative Evidence Synthesis

The conceptual component of this Perspective was based on a narrative rather than systematic review methodology. No prospectively registered protocol, predefined eligibility criteria, exhaustive search strategy, duplicate study selection, or formal study-level risk-of-bias assessment was applied. The included literature was selected according to its relevance to the proposed framework, which creates a possibility of selection and interpretation bias and does not ensure that all relevant or conflicting evidence was captured.

The qualitative classifications of evidence strength presented in Section Critical Appraisal of the Supporting Evidence and Table 1 were based on the consistency, directness, and methodological characteristics of the available literature. They should not be interpreted as a formal GRADE or certainty-of-evidence assessment. The Perspective also combines evidence from meta-analyses, observational studies, mechanistic investigations, and experimental animal models. These study designs address different questions and cannot be considered methodologically equivalent.

### 7.2. Limitations of the Conceptual Framework

The proposed intergenerational GDM–PCOS continuum integrates associations that differ substantially in evidentiary strength. The increased risk of GDM among women with PCOS and the association between intrauterine exposure to maternal diabetes and later offspring metabolic vulnerability are supported by comparatively stronger evidence. In contrast, the direct pathway from maternal GDM exposure to clinically defined PCOS in adult female offspring remains supported primarily by biological plausibility and indirect evidence.

The framework is therefore non-causal, probabilistic, and hypothesis-generating. It does not imply that all women with PCOS will develop GDM, that all offspring exposed to GDM will develop metabolic dysfunction, or that female offspring will subsequently develop PCOS. Shared genetic susceptibility, maternal and paternal metabolic characteristics, body mass index, age, ethnicity, PCOS phenotype, fertility treatment, socioeconomic conditions, lifestyle, healthcare access, and the postnatal environment may confound, mediate, or modify the proposed relationships.

Evidence derived from experimental models should also be interpreted cautiously. Prenatal androgen, anti-Müllerian hormone, or metabolic exposure models provide important mechanistic information but do not fully reproduce the heterogeneous endocrine-metabolic environment, diagnostic criteria, and life-course development of human PCOS. Similarly, epigenetic alterations identified in maternal, placental, or offspring tissues cannot presently be classified with certainty as causes, mediators, consequences, or biomarkers of metabolic dysfunction.

### 7.3. Limitations of the Registry-Based Epidemiological Illustration

The registry component was based on annual aggregated surveillance counts rather than individual-level clinical records. Consequently, record-level completeness and missingness could not be assessed, and ICD-10-coded diagnoses could not be independently validated against oral glucose tolerance test results, laboratory findings, medical records, or treatment information. The extent of under-recording, duplicate recording, diagnostic misclassification, and changes in institutional reporting completeness therefore remains unknown.

The available dataset did not contain information on maternal age, body mass index, parity, ethnicity, socioeconomic status, PCOS diagnosis or phenotype, fertility treatment, gestational weight gain, glucose-testing strategy, treatment exposure, glycemic control, pregnancy outcomes, or offspring health. Adjustment for confounding, stratified analyses, evaluation of effect modification, and direct testing of the proposed GDM–PCOS continuum were therefore not possible.

Annual delivery counts were used as approximate denominators, but the available aggregate sources did not permit confirmation that the diagnostic counts and delivery denominators represented perfectly aligned populations in every calendar year. The resulting values should therefore be interpreted as registry-recorded diagnosis rates rather than estimates of individual-level prevalence or incidence.

Only ten annual observations were available for the 2015–2024 period. No formal time-series analysis, assessment of change points, or quantitative forecasting was undertaken. Interannual differences may reflect changes in screening coverage, diagnostic thresholds, laboratory availability, referral pathways, healthcare utilization, coding procedures, electronic reporting, or institutional data submission rather than true changes in GDM occurrence. Disruptions to antenatal care and modifications of GDM screening during the COVID-19 period may have further influenced diagnostic ascertainment, but the magnitude and direction of this potential bias could not be evaluated.

The registry findings are also context-specific and may not be generalizable to other regions or healthcare systems. Most importantly, because no information on PCOS status or longitudinal offspring outcomes was available, the registry-based illustration cannot empirically validate the proposed intergenerational continuum.

Despite these limitations, the Perspective provides a structured synthesis of current evidence, explicitly distinguishes stronger associations from hypothetical links, and identifies the clinical variables and longitudinal data required to test the proposed framework in future studies.

## 8. Conclusions

Gestational diabetes mellitus (GDM) and polycystic ovary syndrome (PCOS) are clinically distinct disorders that may reflect overlapping metabolic and reproductive vulnerability across different stages of the female life course. The association between PCOS and increased susceptibility to GDM is supported by comparatively strong evidence, while intrauterine exposure to maternal diabetes is consistently associated with later metabolic vulnerability in offspring. In contrast, direct evidence that GDM exposure contributes to clinically defined PCOS in female offspring remains limited and indirect.

The proposed intergenerational GDM–PCOS continuum should therefore be interpreted as a non-causal, probabilistic, and hypothesis-generating framework rather than as an established sequence of disease transmission. Maternal body mass index, age, PCOS phenotype, genetic background, ethnicity, socioeconomic conditions, treatment exposure, healthcare access, and the postnatal environment may modify each stage of the proposed pathway.

The registry-based epidemiological illustration does not validate the conceptual continuum or demonstrate changes in true GDM prevalence. Instead, the observed interannual variability highlights the influence of screening, diagnostic ascertainment, coding, reporting completeness, and healthcare-system organization on the recorded burden of GDM. Standardized and linkable maternal–offspring surveillance data will be required before population-level intergenerational relationships can be evaluated.

The clinical value of the framework lies in connecting established prevention opportunities before conception, during pregnancy, and after delivery. Improved preconception metabolic assessment in women with PCOS, appropriate GDM screening and treatment, postpartum cardiometabolic follow-up, and long-term health promotion for mothers and offspring may reduce cumulative metabolic risk. Prospective longitudinal studies integrating maternal phenotype, metabolic exposures, treatment, placental and epigenetic markers, and offspring reproductive-metabolic outcomes are needed to determine which components of the proposed continuum are causal and clinically actionable.

## Figures and Tables

**Table 2:** Registry-recorded gestational diabetes mellitus diagnoses and delivery denominators, 2015–2024.

Year	GDM Cases, Belgrade	Deliveries, Belgrade	GDM Rate per 1000 Deliveries, Belgrade	GDM Cases, Total Dataset	Deliveries, Serbia	GDM Rate per 1000 Deliveries, Total Dataset
**2015**	282	19,988	14.11	349	64,643	5.40
**2016**	306	19,693	15.54	372	63,855	5.83
**2017**	287	19,289	14.88	343	63,335	5.42
**2018**	396	19,301	20.52	465	62,372	7.46
**2019**	170	19,301	8.81	185	62,372	2.97
**2020**	70	18,869	3.71	91	60,864	1.50
**2021**	62	18,625	3.33	73	59,854	1.22
**2022**	108	19,412	5.56	125	60,556	2.06
**2023**	47	19,139	2.46	57	58,277	0.98
**2024**	64	19,313	3.31	73	58,080	1.26

Note: GDM was identified using ICD-10 code O24.4. Delivery denominators were obtained from the Health Statistical Yearbooks of the Republic of Serbia and corresponding official annual reports. The presented values represent registry-recorded diagnoses per 1000 deliveries and should not be interpreted as individual-level prevalence or incidence estimates. No adjustment was possible for maternal demographic characteristics, metabolic risk factors, screening coverage, diagnostic criteria, coding practices, reporting completeness, or healthcare-system changes.

## Data Availability

The aggregated public health surveillance data supporting the findings of this study are not publicly available due to institutional and legal restrictions. Data may be available from the corresponding author upon reasonable request and with permission of the Belgrade Institute of Public Health.

## Supplementary Materials

The following supporting information can be downloaded at: https://www.mdpi.com/article/10.3390/healthcare14142204/s1, Supplementary File S1: Aggregated registry-recorded gestational diabetes mellitus diagnoses and delivery denominators used for the descriptive registry-based illustration.
